# Effect of combinations of marketed human anthelmintic drugs against *Trichuris muris in vitro* and *in vivo*

**DOI:** 10.1186/1756-3305-5-292

**Published:** 2012-12-11

**Authors:** Jennifer Keiser, Lucienne Tritten, Roberto Adelfio, Mireille Vargas

**Affiliations:** 1 Department of Medical Parasitology and Infection Biology, Swiss Tropical and Public Health Institute, P.O. Box, CH–4002, Basel, Switzerland; 2 University of Basel, Basel, CH–4003, Switzerland

**Keywords:** Combination chemotherapy, *Trichuris muris*, Albendazole, Mebendazole, Pyrantel pamoate, Ivermectin, Levamisole, *In vitro*, *In vivo*

## Abstract

**Background:**

Soil-transmitted helminth (STH) infections are responsible for a huge public health burden, however treatment options are limited. The discovery and development of novel efficacious drugs or drug combinations for the treatment of STH infections therefore has a high research priority.

**Methods:**

We studied drug combination effects using the main standard anthelmintics, albendazole, mebendazole, levamisole, pyrantel pamoate and ivermectin in the *Trichuris muris* model. Drug combinations were first tested *in vitro* and additive and synergistic combinations investigated further *in vivo* in female mice using ratios based on the ED_50_ of the respective drugs.

**Results:**

*In vitro* all 10 combinations of the standard anthelmintics tested against *T. muris* revealed synergistic behavior. We identified three drug combinations *in vivo* as strongly synergistic, namely mebendazole-ivermectin (Combination index (CI)=0.16), mebendazole-levamisole (CI=0.17) and albendazole-mebendazole (CI=0.23). For albendazole-ivermectin, moderate synergism was observed (CI=0.81) and for albendazole-levamisole a nearly additive effect was documented (CI=0.93) *in vivo*. Five combinations (albendazole-pyrantel pamoate, mebendazole-pyrantel pamoate, levamisole-pyrantel pamoate, levamisole-ivermectin and pyrantel pamoate-ivermectin) were antagonistic *in vivo.*

**Conclusion:**

Our results strengthen the evidence that combination chemotherapy might play a role in the treatment of *Trichuris* infections. Albendazole-mebendazole should be studied in greater detail in preclinical studies.

## Background

Soil-transmitted helminth (STH) infections impose a major public health burden mostly among poor populations. It has been estimated that in 2010, 5.3 billion people, of these 1.0 billion school-aged children, were at risk of infection with at least one STH species [1]. Preventive chemotherapy, with the two benzimidazoles, albendazole and mebendazole forms the bedrock of helminth control initiatives preventing morbidity due to helminthiases [2]. However, the number of drugs used to treat infections with STH are limited, have only a low efficacy against *Trichuris trichiura* as a single dose treatment and there is a potential for the development of drug resistance [3,4]. Therefore, recently the Disease Reference Group on Helminth Infections established by the Special Programme for Research and Training in Tropical Diseases (TDR) was given the mandate to develop a research and development agenda for intervention tools considered necessary for control and elimination of human helminthiases [5]. The discovery and development of novel efficacious drugs was identified as one of the top research priorities [6]. In addition, it has been emphasized that more research should be undertaken to investigate whether combinations of different anthelmintics would reveal synergistic effects and would therefore improve control of helminth infections [6]. Indeed, to date, limited research has been done to assess whether combinations of different anthelmintics produce synergistic effects. We have recently studied the effects of oxantel pamoate in combination with standard drugs albendazole, mebendazole, pyrantel pamoate, levamisole and ivermectin against *T. muris in vitro* and *in vivo* (Keiser *et al*., submitted for publication). Using *Caenorhabditis elegans*, significant synergy was documented between Cry proteins from *Bacillus thuringiensis* and tribendimidine or levamisole [7]. Similarly, tribendimidine combined with levamisole showed synergistic effects against *Ancylostoma ceylanicum in vitro* and *in vivo* in male Syrian Golden hamsters [8]. Several clinical trials have also been conducted with drug combinations in patients infected with hookworms or *T. trichiura*[4,9-11], however, the scientific basis for selecting these combinations is not clear. Prior to clinical trials, drug combination studies *in vitro* and/or in animal models followed by preclinical drug interaction studies should be carried out to determine the basis and rationale for studies in humans and to confirm that concomitant use of the medications is safe.

The aim of the present study was to analyze drug combination effects using the main standard anthelmintics albendazole, mebendazole, levamisole, pyrantel pamoate and ivermectin in the *T. muris* mouse model. Drug combinations were first tested *in vitro* and additive and synergistic combinations further investigated *in vivo*.

## Methods

### Drugs

Albendazole and levamisole were purchased from Fluka (Buchs, Switzerland). Mebendazole, ivermectin and pyrantel pamoate were acquired from Sigma-Aldrich (Buchs, Switzerland). Drug stock solutions of 10 mg/ml were prepared in 100% DMSO (Sigma-Aldrich, Buchs, Switzerland) for *in vitro* studies. These were stored at 4°C pending use. For *in vivo* treatments, the drugs were suspended in 10% Tween 80 [70% EtOH (70:30 v/v)] (Buchs, Switzerland) and 90% deionised H_2_O just prior to oral administration.

### Ethics statement

Experiments were performed in accordance with the 3R rules for animal experiments. The current study was approved by the cantonal veterinary office Basel-Stadt (Switzerland) based on Swiss cantonal and national regulations (permission no. 2070).

### Animals and infections

Four week-old female C57BL/10 mice were purchased from Harlan (Blackthorn, UK). Before infection, animals were acclimatized for one week in the animal facility. Groups of 10 mice were kept in macrolon cages with *ad libitum* access to water and rodent food (Rodent Blox from Eberle NAFAG, Gossau, Switzerland). The *T. muris* life-cycle and maintenance have been described elsewhere [12,13]. Briefly, mice were treated with 1 mg/l dexamethasone (dexamethasone-water soluble, Sigma-Aldrich) supplied in the drinking water 2 days before infection by oral gavage with 200 embryonated *T. muris* eggs until 2 days preceding treatment. Adult worms were recovered from the intestines of sacrificed animals from day 35 p.i. onwards (binocular, magnification 16x) and kept at 37°C, 5% CO_2_, in RPMI medium [10.44 g RPMI 1640 (Gibco, Basel, Switzerland) 5 g albumax H (Gibco), 5.94 g HEPES (Sigma-Aldrich) and 2.1 g sodium bicarbonate (Sigma-Aldrich) in 1 l dH_2_O] supplemented with 5% v/v amphotericin B (stock: 250 μg/ml, Sigma-Aldrich) and 1% v/v penicillin-streptomycin (stock: 10,000 U/ml penicillin + 10 mg/ml streptomycin, Sigma-Aldrich).

### *In vitro* drug combination studies

Two adult worms were incubated in each well of a 48-well plate containing 500 μl pre-warmed supplemented RPMI medium (described above), and 500 μl drug solution. IC_50_ (50% inhibitory concentration) values of the standard drugs were determined in our laboratory prior to this work and are summarized in Table 1. In more detail, for levamisole and pyrantel pamoate, IC_50_s of 16.5 and 34.1 μg/ml were reported [13]. If no activity of the drugs on the worms was observed at the highest concentration tested as for albendazole, mebendazole and ivermectin, IC_50_ values of 200 μg/ml were chosen arbitrarily for the drug combination studies presented here. Six concentrations of each drug concentration were tested, starting with the IC_50_ value of each partner drug (IC_50_+IC_50_), which was two-fold diluted up to a final concentration of 1/32IC_50_+1/32IC_50_. Control worms were incubated in medium with equivalent DMSO concentrations (maximum 4% v/v). The plate was incubated at 37°C and 5% CO_2_. After 72 h the motility was evaluated under a light microscope (magnification 20-80x) using a motility scale from 0 to 3 (0 = dead, 1 = very low motility, 2 = low motility, 3 = normal motility) as described previously [13]. Experiments were carried out in duplicate and repeated at least twice. Data obtained from the individual experiments were averaged and adjusted to the values obtained from untreated controls. The nature of each drug combination was characterized by a combination index (CI) as described by Chou [14] and calculated with CompuSyn (CompuSyn, version 3.0.1, ComboSyn, Inc., Paramus, NJ 2007). We used the following classification CI<0.1 very strong synergism, CI: 0.1-0.3 strong synergism, CI: 0.3-0.7 synergism, CI: 0.7-0.85 moderate synergism, CI: 0.85-0.9 slight synergism, CI: 0.9-1.1 nearly additive and CI>1.1 antagonism. Synergistic and additive drug combinations identified *in vitro* (CI≤1) were tested in infected animals using a constant dose ratio as described below.

**Table 1:** **Activity of combinations of standard anthelmintics against *T. muris in vitro, *expressed as IC **_**50**_**values and combination indices (CI)**

**Drug activity against *T. muris *adult worms *in vitro***	
**Single drug**	**IC**_**50**_**s (μg/ml) (r)**
Albendazole	> 200 (0.40)^§^
Mebendazole	> 200 (0.58)
Levamisole	16.5 (1.0)^§^
Pyrantel pamoate	34.1 (0.99)^§^
Ivermectin	> 200 (0.40)
**Drug combination**	**CI at IC**_**50**_
Albendazole-mebendazole	< 0.001
Albendazole-levamisole	0.12
Albendazole-pyrantel pamoate	0.11
Albendazole-ivermectin	< 0.001
Mebendazole-levamisole	0.04
Mebendazole-pyrantel pamoate	0.12
Mebendazole-ivermectin	< 0.001
Levamisole-pyrantel pamoate	0.007
Levamisole-ivermectin	0.12
Pyrantel pamoate-ivermectin	0.14

IC_50_ median inhibitory concentration. r = linear correlation coefficient of the median-effect plot, indicating the goodness of fit. r ≥ 0.85 indicates a satisfactory fit. ^§^ IC_50_s of albendazole, levamisole, and pyrantel pamoate have been published elsewhere [13].

### *In vivo* drug combination studies

On day 40 post-infection the presence of a chronic *T. muris* infection was confirmed in each mouse by an egg positive stool examination. Groups of 4 mice were assigned to treatment or served as untreated control. ED_50_s (median effective doses) of each standard drug had been determined in our laboratory prior to this work and were as follows; 345 mg/kg for albendazole, 79 mg/kg for mebendazole (Keiser *et al*., submitted for publication), 46 mg/kg for levamisole and 4 mg/kg for ivermectin [15]. An arbitrary value of 300 mg/kg was chosen for pyrantel pamoate, for which no ED_50_ could be calculated. The ratio of the ED_50_s of each partner drug was chosen as starting dose (drug 1 ED_50_+ drug 2 ED_50_). If a combination treatment decreased the worm burden by at least 75% (threshold for additivity when the dose effect curves for both drugs are hyperbolic [14]), the drug doses were reduced by half. Stools were collected for up to 72 h post-treatment and the expelled worms were counted. Mice were killed one week post-treatment by exposure to CO_2_, the entire intestine was dissected and all remaining worms counted. Drug activity was expressed by worm burden reduction (WBR) and worm expulsion rate (WER) as described elsewhere [8]. The significance of the WBR was tested using the Kruskal-Wallis test (several treatment doses vs. controls) or the Mann–Whitney U-test (one treatment dose vs. control) using StatsDirect (version 2.4.5; StatsDirect Ltd; Cheshire, UK). Combination indices (CI) were calculated with CompuSyn as described above.

## Results

### Activity of combinations of standard anthelmintics against *T. muris* i*n vitro*

All 10 combinations of the standard anthelmintics tested against *T. muris in vitro* resulted in a synergistic interaction (Table 1). A very strong synergism (CI<0.1) was observed for albendazole-mebendazole, albendazole-ivermectin and mebendazole-ivermectin (all CIs<0.001), mebendazole-levamisole (CI=0.04) and levamisole-pyrantel pamoate (CI=0.007). For the other 5 combinations (albendazole-levamisole, albendazole-pyrantel pamoate, mebendazole-pyrantel pamoate, levamisole-ivermectin and pyrantel pamoate-ivermectin a strong synergism (CI=0.11-0.14) was determined.

### Activity of combinations of standard anthelmintics against *T. muris in vivo*

Since all anthelmintic drug combinations yielded synergistic effects *in vitro*, they were further investigated *in vivo* using the ED_50_ of each drug (drug 1 ED_50_ + drug 2 ED_50_) as starting dose. The results are summarized in Table 2. We identified three drug combinations as strongly synergistic, namely mebendazole-ivermectin (CI=0.16), mebendazole-levamisole (CI=0.17) and albendazole-mebendazole (CI=0.23). These drug combinations still achieved significant WBRs when drug dosages of 1/16 ED_50_ were combined. For example, a WBR of 70.3% was achieved, when 4.9 mg/kg mebendazole was combined with 0.25 mg/kg ivermectin. Note that mebendazole plus ivermectin (at the highest dose) was the only combination that resulted in complete elimination of worms.

**Table 2:** **Worm expulsion rates, worm burden reductions and corresponding combination index values obtained following treatment of mice harboring adult*T. muris*with combinations of standard anthelmintics**

**Group**	**Dose (mg/kg)**	**Mean number of worms (SD)**	**Mean number of expelled worms (SD)**	**Worm expulsion rate (%)**	**Worm burden reduction (%)**	***P*-value**	**Combination index at ED**_**50**_
Control 1	–	93.25 (9.46)	0.75 (0.96)	0.8	–	–	–
Control 2	–	123.25 (35.07)	0.25 (0.50)	0.2	–	–	–
Control 3	–	91.25 (23.73)	0 (0)	0	–	–	–
Control 4	–	78.33 (20.79)	0 (0)	0	–	–	–
Control 5	–	85.71 (21.75)	0 (0)	0	–	–	–
Control 6	–	82.57 (30.59)	0 (0)	0	–	–	–
Control 7	–	94.4 (39.21)	0 (0)	0	–	–	–
Control 8	–	56.50 (22.07)	0 (0)	0	–	–	–
ABZ-MBZ	345+79^3^	77.50 (63.07)	60.50 (55.87)	78.06	81.37	<0.001^a^	0.23
	172.5+39.5^4^	73.25 (102.66)	62.75 (91.34)	85.67	86.60		
	86.25+19.75^7^	95.0 (5.66)	63.0 (1.41)	66.32	66.10		
	43.125+ 9.875^8^	77.75 (33.54)	47.75 (22.34)	61.41	46.90		
ABZ-LEV	345+46^5^	117.50 (112.86)	107.0 (112.75)	91.06	87.75	0.001^a^	0.93
	172.5+23^7^	89.25 (42.0)	46.50 (35.61)	52.10	54.71		
ABZ-PYR	345+300^4^	63.5 (41.30)	19.75 (11.76)	31.10	44.15	0.229^b^	–
ABZ-IVM	345+4^2^	136.50 (14.98)	109.50 (15.76)	80.22	78.05	0.003^a^	0.81
	172.5+2^7^	80.50 (17.06)	41.50 (9.26)	51.55	58.69		
MBZ-LEV	79+46^1^	86.0 (53.20)	82.75 (48.11)	96.22	96.49	<0.001^a^	0.17
	39.5+23^2^	82.0 (17.87)	74.5 (21.32)	90.85	93.90		
	19.75+12.5^3^	96.25 (37.25)	81.25 (39.10)	84.42	83.56		
	9.875+6.25^4^	61.25 (33.98)	50.50 (37.36)	82.45	86.28		
	4.94+3.125^7^	125.50 (71.01)	48.75 (37.55)	38.84	18.70		
MBZ-PYR	79+300^4^	41.25 (24.65)	38.75 (24.92)	93.94	96.81	0.049^a^	N.D.
	39.5+150^8^	161.75 (98.22)	113.75 (77.82)	70.32	15.04		
MBZ-IVM	79+4^2^	92.50 (26.71)	92.50 (26.71)	100	100	<0.001^a^	0.16
	39.5+2^2^	70.33 (32.04)	68.0 (32.19)	96.68	98.10		
	19.75+1^3^	46.25 (18.23)	36.75 (11.53)	79.46	89.59		
	9.875+0.5^4^	54.0 (44.74)	41.50 (36.74)	76.85	84.04		
	4.94+0.25^7^	101.0 (57.30)	73.0 (34.70)	72.28	70.34		
	2.47+0.125^8^	70.33 (26.86)	16.67 (22.85)	23.70	5.01		
LEV-PYR	46+300^6^	46.25 (18.84)	29.50 (19.28)	63.78	79.71	0.057^a^	N.D.
	23+150^8^	87.75 (57.34)	10.50 (10.88)	11.97	0		
LEV-IVM	46+4^4^	66.25 (42.84)	59.0 (42.78)	89.06	90.74	0.009^a^	1.38
	23+2^8^	60.0 (24.51)	14.50 (13.80)	24.17	19.47		
PYR-IVM	300+4^4^	52.25 (36.04)	46.75 (32.71)	89.47	92.98	0.032^a^	N.D.
	150+2^8^	122.50 (112.38)	32.75 (17.39)	26.73	0		

Numbers in superscript refer to the corresponding control group. ^a^*P*-values were obtained from the Kruskal-Wallis test (several treatment doses vs. controls), ^b^*P*-values were obtained from the Mann–Whitney U test (one treatment dose vs. controls). The CI at ED_50_ are based on worm burden reductions. N.D. = not determined. ABZ: albendazole; MBZ: mebendazole; LEV: levamisole; PYR: pyrantel pamoate; IVM: ivermectin.

For albendazole plus ivermectin a moderate synergism was observed (CI=0.81) and when albendazole was administered together with levamisole a nearly additive effect was documented (CI=0.93).

Five combinations (albendazole-pyrantel pamoate, mebendazole-pyrantel pamoate, levamisole-pyrantel pamoate, levamisole-ivermectin and pyrantel pamoate-ivermectin) revealed an antagonistic behavior. Four of these drug combinations (mebendazole-pyrantel pamoate, levamisole-pyrantel pamoate, levamisole-ivermectin and pyrantel pamoate-ivermectin) showed an effect at the highest dosage tested (ED_50_+ED_50_) with WBRs of 79.7-96.8% but only low activity when half of these dosages were used. Albendazole-pyrantel pamoate (ED_50_+ED_50_) resulted in only a moderate worm burden reduction of 44.2%.

## Discussion

The treatment of *T. trichiura* infections in humans has significant deficiencies, since none of the available anthelmintics achieves high cure rates and egg reduction rates, when the drugs are administered as single doses in the framework of preventive chemotherapy treatment campaigns [2,3]. Combination chemotherapy is common medical practice in several medical fields such as cancer, bacterial infections, HIV or malaria [4,16,17] as well as in veterinary medicine [18] since an efficacious intervention often requires the combined action of two or more interacting therapeutic components. Furthermore, avoiding drug resistance has been a major rationale for combination chemotherapy from the start [16,17].

**Figure 1:**
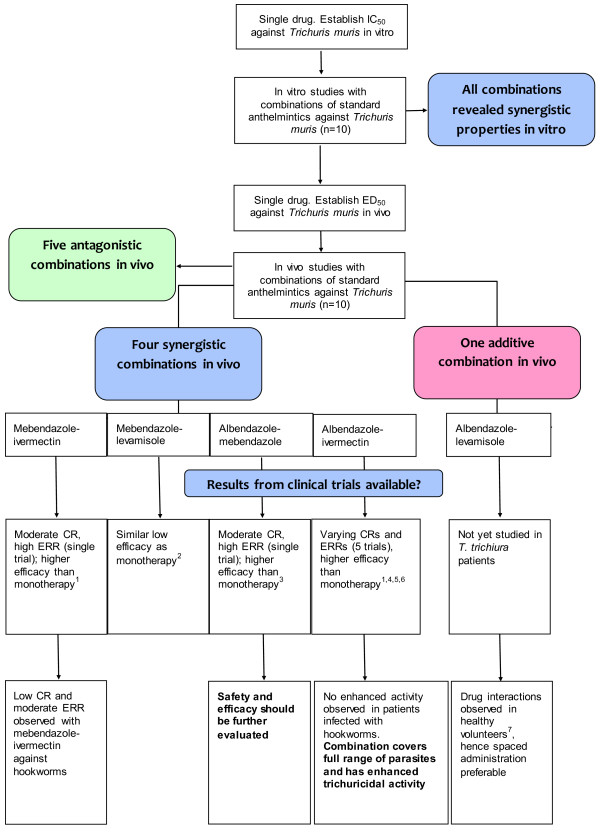
**Study flow diagram, illustrating the results of our studies and placing them in context with clinical trials.**^1^: Reference [10], ^2^ Reference [22], ^3^ Reference [11], ^4^ Reference [24], ^5^ Reference [25], ^6^ Reference [26], ^7^ Reference [27]. ERR: egg reduction rate; CR: cure rate.

To the best of our knowledge, we have for the first time thoroughly evaluated the activity of combinations of available marketed human anthelmintics against the laboratory model *T. muris in vitro* and *in vivo*. *In vitro,* all combinations were found to be synergistic (Figure 1) and therefore followed up *in vivo*. Four drug combinations were identified *in vivo* using drug doses based on ED_50_ values, which showed synergistic properties namely mebendazole-ivermectin, mebendazole-levamisole, albendazole-mebendazole and albendazole plus ivermectin. In addition, for the combination of albendazole and levamisole an additive trichuricidal effect was documented (Figure 1). A good correlation between the *in vitro* and *in vivo* results was observed with the 3 drug combinations showing a strongly synergistic effect *in vitro* (albendazole-mebendazole, albendazole-ivermectin and mebendazole-ivermectin) also revealing synergism *in vivo*. It is interesting to note that the synergistic combinations identified comprise at least one benzimidazole. Drug combinations containing pyrantel pamoate as well as levamisole combined with ivermectin were found to be antagonistic. An increased bioavailability of ivermectin was observed when the drug was given concurrently with levamisole in healthy volunteers [19], however, it is not clear whether this pharmacokinetic interaction has an influence on the activity against *T. muris* in mice. Unfortunately, possible mechanisms underlying synergistic or antagonistic effects are presently not available for any of these anthelmintic drug combinations. It has recently been suggested that an increased efficacy of an albendazole-mebendazole combination over albendazole or mebendazole monotherapy might be explained by an extended contact period of the worms with the active compounds (active compounds present successively: mebendazole first followed by the main metabolite of albendazole, albendazole sulfoxide [11]). However, it has been suggested that the parent drug albendazole, more lipophilic, exerts the main anthelmintic action as the transcuticular diffusion is thought to be more important for drug uptake by worms [20,21], which would contradict the above mentioned hypothesis of an increased activity due to albendazole sulfoxide.

Clinical trials have been conducted with the four synergistic combinations reported here (Figure 1). Ironically these trials have been conducted without any preclinical supporting information, either on efficacy or potential toxicity. No enhanced trichuricidal efficacy was observed when a combination of mebendazole and levamisole was given to school-aged children on Pemba island [22]. However, our findings of the synergistic effect of albendazole-ivermectin, mebendazole-ivermectin and albendazole-mebendazole in *T. muris*-infected mice are in line with results from patients infected with *T. trichiura*. Briefly, five studies investigated the efficacy of albendazole-ivermectin [10,23-26] and increased cure rates and egg reduction rates for the combination compared to albendazole or ivermectin were observed in all trials. Clearly, a tremendous advantage of an albendazole-ivermectin combination is its broad spectrum of activity targeting not only the soil-transmitted helminths, including *Strongyloides stercoralis* but also lymphatic filariasis and onchocerciasis. In three of the trials the effect of albendazole-ivermectin was also assessed against infections with hookworms, however, no increased therapeutic benefit was observed for albendazole-ivermectin over albendazole [10,22,24]. Moreover, the combination has already been studied in onchocerciasis patients and no pharmacokinetic interactions were observed [19]. Mebendazole-ivermectin showed an even superior activity to albendazole-ivermectin [10], which might be explained by the higher sensitivity of *Trichuris* spp. to mebendazole than albendazole. However, it is important to mention that the combination of mebendazole and ivermectin only achieved moderate egg reduction rates and low cure rates against hookworm infections [10]. Finally, albendazole-mebendazole achieved a higher cure rate (46.1%) compared to albendazole (6.0%) and mebendazole (11%) in school-aged children infected with *T. trichiura* in Uganda [11].

In the present work an additive effect was observed when levamisole was administered in combination with albendazole to *T. muris*-infected mice. To our knowledge, this combination has not been used in the treatment of trichuriasis. However, Awadzi and colleagues have shown that clinically significant drug interactions occur between the two drugs, resulting in a reduction of albendazole sulfoxide and levamisole plasma levels as well as unexpected adverse events in onchocerciasis patients [27]. Hence, given these pharmacokinetic interactions a simultaneous co-administration of albendazole and levamisole cannot be recommended.

## Conclusions

We have identified four synergistic and one additive combination against *T. muris* in the mouse model. To shed light on the next steps to be undertaken we have placed our findings in context with Phase I and Phase II clinical trials conducted with these drug combinations in previous years. In addition, it has to be emphasized that the four species of nematodes (*Ascaris lumbricoides*, *T. trichiura* and both hookworm species, *Ancylostoma duodenale* and *Necator americanus*) are treated collectively in preventive chemotherapy programs and the treatments should therefore have high activities against all these species. Albendazole-mebendazole has emerged as an interesting and unexpected drug combination, hence this treatment should be studied in greater detail including drug interaction studies as well as studies against hookworms. In addition, albendazole-ivermectin offers the advantage that it is well studied, has increased trichuricidal activity over albendazole and ivermectin alone and has a broad spectrum of activity.

## Competing interests

The authors declare that they have no competing interests.

## Authors’ contributions

JK and LT designed the studies. MV and RA carried out the experiments. JK and LT wrote the manuscript. All authors read and approved the final version of the manuscript.

## References

[B1] Pullan RL Brooker SJ The global limits and population at risk of soil-transmitted helminth infections in 2010 Parasit Vectors 2012 5 81 10.1186/1756-3305-5-81 22537799 PMC3419672

[B2] WHO Preventive chemotherapy in human helminthiasis: coordinated use of anthelminthic drugs in control interventions: a manual for health professionals and programme managers 2006 Geneva: World Health Organization

[B3] Keiser J Utzinger J Efficacy of current drugs against soil-transmitted helminth infections: systematic review and meta-analysis JAMA 2008 299 1937 1948 18430913 10.1001/jama.299.16.1937

[B4] Keiser J Utzinger J The drugs we have and the drugs we need against major helminth infections Adv Parasitol 2010 73 197 230 20627144 10.1016/S0065-308X(10)73008-6

[B5] Utzinger J A research and development agenda for the control and elimination of human helminthiases PLoS Negl Trop Dis 2012 6 e1646 10.1371/journal.pntd.0001646 22545174 PMC3335882

[B6] Prichard RK Basanez MG Boatin BA McCarthy JS Garcia HH Yang GJ Sripa B Lustigman S A research agenda for helminth diseases of humans: intervention for control and elimination PLoS Negl Trop Dis 2012 6 e1549 10.1371/journal.pntd.0001549 22545163 PMC3335868

[B7] Hu Y Platzer EG Bellier A Aroian RV Discovery of a highly synergistic anthelmintic combination that shows mutual hypersusceptibility Proc Natl Acad Sci USA 2010 107 5955 5960 10.1073/pnas.0912327107 20231450 PMC2851895

[B8] Tritten L Nwosu U Vargas M Keiser J In vitro and in vivo efficacy of tribendimidine and its metabolites alone and in combination against the hookworms Heligmosomoides bakeri and Ancylostoma ceylanicum Acta Trop 2012 122 101 107 10.1016/j.actatropica.2011.12.008 22210439

[B9] Geary TG Woo K McCarthy JS Mackenzie CD Horton J Prichard RK de Silva NR Olliaro PL Lazdins-Helds JK Engels DA Unresolved issues in anthelmintic pharmacology for helminthiases of humans Int J Parasitol 2010 40 1 13 10.1016/j.ijpara.2009.11.001 19932111

[B10] Knopp S Mohammed KA Speich B Hattendorf J Khamis IS Khamis AN Stothard JR Rollinson D Marti H Utzinger J Albendazole and mebendazole administered alone or in combination with ivermectin against Trichuris trichiura: a randomized controlled trial Clin Infect Dis 2011 51 1420 1428 21062129 10.1086/657310

[B11] Namwanje H Kabatereine NB Olsen A Efficacy of single and double doses of albendazole and mebendazole alone and in combination in the treatment of Trichuris trichiura in school-age children in Uganda Trans R Soc Trop Med Hyg 2011 105 586 590 10.1016/j.trstmh.2011.07.009 21885077

[B12] Wakelin D The stimulation of immunity and the induction of unresponsiveness to Trichuris muris in various strains of laboratory mice Z Parasitenkd 1970 35 162 168 5534152 10.1007/BF00259993

[B13] Tritten L Silbereisen A Keiser J In vitro and in vivo efficacy of monepantel (AAD 1566) against laboratory models of human intestinal nematode infections PLoS Negl Trop Dis 2011 5 e1457 10.1371/journal.pntd.0001457 22216366 PMC3246443

[B14] Chou TC Drug combination studies and their synergy quantification using the Chou-Talalay method Cancer Res 2010 70 440 446 10.1158/0008-5472.CAN-09-1947 20068163

[B15] Tritten L Silbereisen A Keiser J Nitazoxanide: In vitro and in vivo drug effects againstTrichuris murisandAncylostoma ceylanicum, alone or in combination Int J Parasitol: Drugs and Drug Resistance 2012 2 98 105 10.1016/j.ijpddr.2012.02.004 PMC3862390 24533270

[B16] White N Antimalarial drug resistance and combination chemotherapy Philos Trans R Soc Lond B Biol Sci 1999 354 739 749 10.1098/rstb.1999.0426 10365399 PMC1692562

[B17] Jia J Zhu F Ma X Cao Z Li Y Chen YZ Mechanisms of drug combinations: interaction and network perspectives Nat Rev Drug Discov 2009 8 111 128 10.1038/nrd2683 19180105

[B18] Bartram DJ Leathwick DM Taylor MA Geurden T Maeder SJ The role of combination anthelmintic formulations in the sustainable control of sheep nematodes Vet Parasitol 2012 186 151 158 10.1016/j.vetpar.2011.11.030 22245073

[B19] Awadzi K Edwards G Duke BO Opoku NO Attah SK Addy ET Ardrey AE Quartey BT The co-administration of ivermectin and albendazole–safety, pharmacokinetics and efficacy against Onchocerca volvulus Ann Trop Med Parasitol 2003 97 165 178 10.1179/000349803235001697 12803872

[B20] Alvarez LI Mottier ML Sanchez SF Lanusse CE Ex vivo diffusion of albendazole and its sulfoxide metabolite into Ascaris suum and Fasciola hepatica Parasitol Res 2001 87 929 934 11728018 10.1007/s004360100471

[B21] Geary TG Sangster NC Thompson DP Frontiers in anthelmintic pharmacology Vet Parasitol 1999 84 275 295 10.1016/S0304-4017(99)00042-4 10456419

[B22] Albonico M Bickle Q Ramsan M Montresor A Savioli L Taylor M Efficacy of mebendazole and levamisole alone or in combination against intestinal nematode infections after repeated targeted mebendazole treatment in Zanzibar Bull World Health Organ 2003 81 343 352 12856052 PMC2572452

[B23] Ndyomugyenyi R Kabatereine N Olsen A Magnussen P Efficacy of ivermectin and albendazole alone and in combination for treatment of soil-transmitted helminths in pregnancy and adverse events: a randomized open label controlled intervention trial in Masindi district, western Uganda AmJTrop Med Hyg 2008 79 856 863 19052293

[B24] Beach MJ Streit TG Addiss DG Prospere R Roberts JM Lammie PJ Assessment of combined ivermectin and albendazole for treatment of intestinal helminth and Wuchereria bancrofti infections in Haitian schoolchildren AmJTrop Med Hyg 1999 60 479 486 10.4269/ajtmh.1999.60.479 10466981

[B25] Ismail MM Jayakody RL Efficacy of albendazole and its combinations with ivermectin or diethylcarbamazine (DEC) in the treatment of Trichuris trichiura infections in Sri Lanka Ann Trop Med Parasitol 1999 93 501 504 10.1080/00034989958230 10690245

[B26] Belizario VY Amarillo ME De Leon WU Reyes AE Dl Bugayong MG Macatangay BJC A comparison of the efficacy of single doses of albendazole, ivermectin, and diethylcarbamazine alone or in combinations against Ascaris and Trichuris spp Bull World Health Organ 2003 81 35 42 12640474 PMC2572315

[B27] Awadzi K Edwards G Opoku NO Ardrey AE Favager S Addy ET Attah SK Yamuah LK Quartey BT The safety, tolerability and pharmacokinetics of levamisole alone, levamisole plus ivermectin, and levamisole plus albendazole, and their efficacy against Onchocerca volvulus Ann Trop Med Parasitol 2004 98 595 614 10.1179/000349804225021370 15324466

